# The Influence of Incentive-Based Mobile Fitness Apps on Users’ Continuance Intention With Gender Moderation Effects: Quantitative and Qualitative Study

**DOI:** 10.2196/50957

**Published:** 2024-06-05

**Authors:** Aaya Faizah, Alifah Fatimah Azzahra Hardian, Rania Devina Nandini, Putu Wuri Handayani, Nabila Cyldea Harahap

**Affiliations:** 1 Faculty of Computer Science, University of Indonesia Depok Indonesia

**Keywords:** incentive, fitness, mobile fitness apps, gender, continuance usage intention, Indonesia, mobile phone

## Abstract

**Background:**

A survey conducted by McKinsey & Company reported that, as of May 2022, as many as 26% of Indonesians had recently started to engage actively in physical activity, 32% undertook regular physical activity, and 9% exercised intensely. The Fourth Industrial Revolution has spurred the rapid development of mobile fitness apps (MFAs) used to track people’s sports activities. However, public interest in using these apps for any length of time is still relatively low.

**Objective:**

In this study, we aimed to determine the effect of incentives (eg, self-monitoring, social support, platform rewards, and external influence) on the use of MFAs and the moderating effect of gender on users’ continuance usage intention.

**Methods:**

The study used a mixed methods approach. Quantitative data were collected through a web-based questionnaire and qualitative data from interviews with 30 respondents. The quantitative data, collected from 379 valid responses, were processed using covariance-based structural equation modeling. The qualitative data were processed using thematic analysis. The MFAs included in this research were those used as sports or physical activity trackers, such as Apple Fitness, Strava, Nike Run Club, and Fita.

**Results:**

The results of the data analysis show that 3 groups of incentives, namely, self-monitoring, platform rewards, and external influence (with the exception of social support), affect the perceived usefulness of these apps. Gender was also shown to moderate user behavior in relation to physical activity. The study showed that women were more likely to be motivated to exercise by social and external factors, while men paid greater attention to the tracking features of the app and to challenges and rewards.

**Conclusions:**

This research contributes to the field of health promotion by providing guidance for MFA developers.

## Introduction

### Background

According to the World Health Organization [1], regular physical activity (PA) is a key factor in the prevention and management of noncommunicable diseases. The Global Status Report on Physical Activity [2] reported that 1.4 billion individuals aged >18 years do not meet the levels of PA recommended to promote and protect health. In 2016, it was reported that, globally, 23% of all men and 32% of all women aged ≥18 years were not sufficiently physically active to stay healthy [1]. This means that approximately 1 in 3 women and 1 in 4 men are not sufficiently active and do not meet the global recommendation of at least 150 minutes of moderate-intensity PA or 75 minutes of high-intensity PA per week [3]. In August 2022, McKinsey & Company released the results of a survey conducted with 1041 Indonesian respondents in which 26% of the respondents stated that they had started to engage actively in personal training, 32% reported that they had been playing sports regularly, and 9% indicated that they had increased the intensity of their sports or fitness activities [4]. These data indicate that the level of Indonesians’ interest in, and awareness of, sports, fitness, and personal training is significantly higher than the global average. This conclusion is supported by the increased use of mobile fitness apps (MFAs) in Indonesia, with 29 million users in 2022 [5].

MFAs use several types of incentives, which include self-incentives, peer incentives, and platform incentives [6-9]. In the MFA context, self-incentives involve a self-monitoring (SM) system in which users monitor and track their own behavior [10,11]. Peer incentives are focused on social support (SS), which includes informational, emotional, and material support or the protection provided by fellow users of the app [12]. In the context of MFAs, platform incentives usually take the form of rewards or awards resulting from gamification features [13]. Users who collect a large number of rewards are usually considered to have a higher status on the MFA and feel more satisfied with their use of the app [14,15].

According to Zhu et al [16], very few studies have examined the role played by gender differences in the use of health and fitness apps. Yin et al [17] stated that achievements in sports motivate men more, while social relationships motivate women more. Previous research on MFAs has explored their design [18] and evaluation [19-22], as well as user adoption intentions [23]. In addition, several studies have discussed continuity in the use of MFAs [24-27]. Chiu et al [25] integrated the expectation-confirmation theory (ECT) with the investment model to analyze the continuous use of MFAs. However, research investigating the various types of MFA incentives has been shown to have several limitations [17,26] because the effects of each incentive have mostly been explored separately [28-30]. Per McKinsey & Company’s 2022 survey among Indonesian citizens [4], 87% of the respondents intended to continue using their personal training and fitness apps. The market analysis and demographics of this study apply only to MFA users in Indonesia.

### Research Question

This research adopted the self-determination theory (SDT) and the ECT. The SDT, as postulated by Ryan and Deci [31], states that there are 3 main psychological needs that drive human behavior: autonomy, relatedness, and competency. If these psychological needs are met, intrinsic motivation will increase and make it easier to maintain certain behaviors [31]. Teixeira et al [32] show that the SDT can be applied to behavioral interventions that relate to exercise or PA. While the SDT has the ability to predict the intensity of a behavior based on the influence of incentive factors [17], the ECT is generally used to predict the continuity of a behavior [25]. The combination of the SDT and the ECT was chosen to analyze the relationship between the incentive factors that affect the use of MFAs and continuity in using them. Thus, the research question is “How do the incentives promoted by MFAs influence users’ continuance usage intention (CUI)?” This research can provide guidance for MFA developers by helping them to evaluate their apps.

## Methods

### Research Model

#### Overview

**Figure 1:**
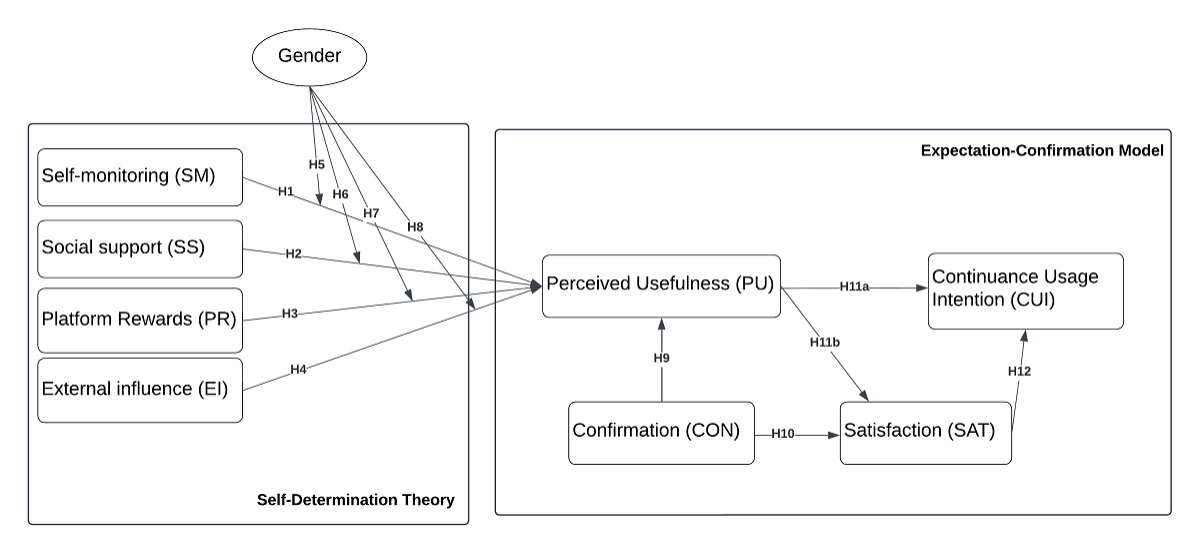
Proposed conceptual model.

The model used for this research is based on 2 theories and 1 moderating effect, namely, the SDT and the ECT, with the moderating effect of gender. Significant studies reporting on the use of these 2 theories include those by Yin et al [17], Huang and Ren [26], Chiu et al [25], and Li et al [33]. Yin et al [17] found that incentives are compatible with the SDT in motivating users’ PA behaviors. The SDT approach described by Yin et al [17] is the theoretical basis for this research because it analyzes incentives offered by MFAs collectively and uses gender as a moderating variable. The relationship between perceived usefulness (PU) and incentives was also analyzed by Huang and Ren [26]. This research suggests that technology functions in MFAs, such as SM, self-regulation, and goal attainment, have an indirect effect on CUI through PU; for instance, Chiu et al [25] and Li et al [33] found that users’ CUI was significantly predicted by ECT. Our research model, which includes 9 variables and 13 hypotheses (described in the following subsections), is presented in Figure 1.

#### The Influence of SM on PU

SM, which is classified as one of the self-incentives in MFAs, includes managing and tracking one’s own behavior [17]. These actions enable users to observe their own progress and evaluate their performance against previously set goals [34]. PU refers to the extent to which a person feels that technology can improve their performance of certain tasks [35]. In this study, the task was identified as increasing the user’s PA, while, for MFA users, PU implies that using the MFA will enhance their personal training intensity [36,37]. Bhattacherjee [38] argues that when users confirm their initial expectations of the main functionality of a mobile app, they will begin to perceive the app as useful for improving their task performance and thus continue to use it. Huang and Ren [26] measured PU relating to the effectiveness and performance of PA through the use of 4 technological functions of the MFA, one of which is SM. Therefore, we examine the following hypothesis:

- H1: SM has an influence on PU.

#### The Influence of SS on PU

SS is classified as one of the peer incentives in MFAs [17]. Web-based SS is seen as an important factor affecting the physical and mental health of individuals, such as sports activity and increased well-being [12,39]. Humans have a tendency to behave in ways that are consistent with people in their own social networks, and this can be exploited in the context of mobile health (mHealth) [29]. Chen and Pu [40] conducted research on social incentives by developing the HealthyTogether mobile game, which allows users to participate in PA together and send messages to one another. The authors showed that users significantly increased their PA when using HealthyTogether compared to when they were exercising alone [40]. Edney et al [6] built the Active Team app, which is an MFA with social and gamification functions. The primary outcome of their study was a change in the total daily minutes of moderate to vigorous PA at 3 months, as measured objectively using an accelerometer [6]. Therefore, we propose the following hypothesis:

- H2: SS has an influence on PU.

#### The Influence of Platform Rewards on PU

The platform rewards include gamification elements, such as badges, points, and leaderboards [17]. The gamification element in MFAs can provide two types of information: (1) the user’s PA progress and (2) a comparison of the user’s PA with that of other users [41]. From this information, MFA users can observe their progress and experience greater satisfaction as they recognize their own personal training achievements. This leads to higher user competency satisfaction and increased behavioral motivation [13,42]. Yin et al [17] found that platform rewards have a positive relationship with users’ PA. This finding is supported by Plangger et al [13] and Huang and Ren [26], who analyzed the effect of the goal-attainment technology function of MFAs, in which users can set their own goals, which are then achieved by undertaking PA. These achievements are then categorized as platform rewards. Huang and Ren [26] also found that this technology function had a positive effect on PU. Therefore, we propose the following hypothesis:

- H3: Platform rewards have a positive influence on PU.

#### The Influence of External Influence on PU

External influence (EI) is one of the extrinsic motivations identified in the SDT, which means that behavior is motivated through influences that do not depend on internal factors [43]. Huang [28] proposed this variable to explain how PA can be promoted through external factors. One example is companies providing incentives to MFA users as part of their corporate social responsibility initiatives [28]. Several studies discuss EI and PA. One example of EI referred to in this study is the name or *image* of a sponsor of an activity [44]. Low and Pyun [45] explain that sponsorship that gives a good impression to customers or users will produce behavior that tends to be positive. In the context of sporting activities, Huang [28] explains that sponsor characteristics play an important role in participation in a sporting activity. Therefore, because an MFA is a tool that can measure a person’s PA, we intend to explore the following hypothesis:

- H4: EI has an influence on PU.

#### The Influence of Gender on SM and PU

According to Mao et al [7], MFA incentives are not always equally effective for women and men. This is because women and men have different ways of thinking [17]. Yin et al [17] conducted research that assumed that gender would influence the effectiveness of SM incentives, making them more effective for men than for women. This assumption was based on the belief that men generally pay more attention to their own achievements than women [46]. Surprisingly, Yin et al [17] show that gender does not affect the effectiveness of SM in MFAs. This finding relates to the concept of self-regulation, which is strongly driven by self-efficacy [47]. Individuals who decide to use MFAs are generally believed to have high self-efficacy in carrying out PA [17]. Therefore, we plan to test the following hypothesis:

- H5: Gender influences the relationship between SM and PU in MFA users.

#### The Influence of Gender on SS and PU

With regard to SS, Yin et al [17] explain that SS is one of the factors that most helps to fulfill the relatedness needs described in the SDT. According to Wang et al [9], social ties and commitment are more important for women than for men in shaping their attitudes toward the sharing of information. In considering gender, Yin et al [17] found that women tend to be more influenced by their relatedness needs than men. Women are also believed to be driven more by collective goals, such as pleasure or interpersonal harmony [48,49]. In the context of health apps, Kimbrough et al [50] found that women are usually more affected by environmental conditions and social relationships than men. Thus, we propose the following hypothesis:

- H6: Gender influences the relationship between SS and PU among MFA users.

#### The Influence of Gender on Platform Rewards and PU

Men tend to focus more on themselves and tend to be more independent than women [46,51]. Men also tend to focus more on completing or achieving individual goals that demonstrate their performance and abilities [46,51]. Relatedly, Vilela and Nelson [52] showed that men tend to be more motivated by their own achievements than women when using information system products. This is due to the general behavioral characteristics of men, who are generally more aggressive, pragmatic, and self-oriented in their behavior compared to women [52]. When specifically applied to incentives and CUI, Yin et al [17] also found an influence between gender and the effectiveness of platform reward incentives. The authors assumed that this is caused by the behavioral characteristics of men, who generally make decisions more rationally and pay greater attention to their own behavior. Thus, we propose the following hypothesis:

- H7: Gender influences the relationship between platform rewards and PU among MFA users.

#### The Influence of Gender on EI and PU

Sun and Zhang [53] state that women have a higher awareness of the environment than men. Leong et al [54] and Li et al [33] also found that men tend to be less easily influenced by external advice or support. Similarly, Venkatesh et al [55] concluded that women tend to be more influenced by EI, while men are usually less affected by external facilitation in their use of technology. This was confirmed by Weman Josefsson et al [56], who showed that men participate in challenges organized by the community to compete, while women participate for social and autonomy reasons. Hence, we propose the following hypothesis:

- H8: Gender influences the relationship between EI and PU among MFA users.

#### The Influence of Confirmation of Expectations on PU

Confirmation of expectations refers to the perceived level of conformity between the information system product or service expectation and actual performance [38]. Bhattacherjee [38] explains that PU refers to the individual’s perception of the anticipated benefits from the use of IT products or services. The ECT implies that the confirmation of a user’s expectations has a positive effect on their perception of the PU of an IT product or service [25,57-59]. According to the cognitive dissonance theory [60], IT users may experience psychological conflict if their initial expectations are not confirmed by their actual use experience [61]. Conversely, if users’ initial expectations are confirmed or met, they may display higher investment behavior and reduce their preference for alternative apps [25]. Hsu and Lin [62] state that confirmation of expectations is positively related to the perceived quality of the IT product or service used, with the result that users tend to ignore quality alternatives. Therefore, we propose the following hypothesis:

- H9: Confirmation of expectations has an influence on PU.

#### The Influence of Confirmation of Expectations on Satisfaction

Chiu et al [25] proposed that confirmation of user expectations affects satisfaction with the app as well as its PU. Satisfaction can be interpreted as an individual’s evaluation of their initial experience with a product or service [38]. Chiu et al [25] explain that before downloading an app, users generally have expectations of it, based on detailed information received from the app provider and on ratings and reviews from other users. After using the app, the user gains experience and evaluates the performance of the app based on previously established expectations. In line with the expectation-confirmation model, Chiu et al [25] assume that users’ perceptions of postuse benefits and the confirmation of previous expectations determine their satisfaction in using IT products and services. Therefore, we propose the following hypothesis:

- H10: Confirmation of expectations has an influence on satisfaction.

#### The Influence of PU on CUI

PU refers to the user’s perception of the benefits expected from using an IT product or service [61,63]. According to Bhattacherjee [38], expectations based on the user’s direct experience have an important role in forming their IT CUI. Chiu et al [25] state that many studies conducted in various contexts [59,64,65] empirically support a positive relationship between PU and CUI. Wu et al [66] show that when users find the mHealth app useful, they show a higher level of satisfaction and tend to use it continuously. Thus, we define the following hypothesis:

- H11a: PU has an influence on CUI.

#### The Influence of PU on Satisfaction

According to Chiu et al [25], PU also has a strong and positive impact on satisfaction. The authors state that the more benefits users receive from health and fitness apps, the greater their satisfaction [25]. When a user has used an app for an extended period of time, the user will evaluate its performance and form either a confirmation or a disconfirmation of judgment with regard to their expectations [62]. Disconfirmation of expectations affects user satisfaction and creates negative perceptions of the usefulness of MFAs. Conversely, users’ positive perceptions of usefulness increase their satisfaction with an app. Therefore, we propose the following hypothesis:

- H11b: PU has an influence on satisfaction.

#### The Influence of Satisfaction on CUI

Satisfaction can be identified as a significant factor influencing consumer behavior [25]. Bhattacherjee [61] strengthens this definition by explaining that user satisfaction is an important determinant of postadoption behavior relating to IT products and services. In other words, users with higher levels of satisfaction will exhibit greater levels of use of IT products and services than those who are less satisfied [25]. Wu et al [66] confirm that satisfied users are more likely to continue using an app because dissatisfied users can easily switch to other technologies at no additional cost. The relationship between satisfaction and CUI has been identified as one of the strongest relationships in the expectation-confirmation model [63]. Therefore, we propose the following hypothesis:

- H12: Satisfaction has an influence on the CUI of MFA users.

### Research Procedure

This study used a mixed methods approach that integrated a quantitative approach, based on a questionnaire, with a qualitative approach, using interviews. The only inclusion criterion for respondents in this study was that they used MFAs. We modified a questionnaire that has been established in previous studies [12,15,16,24,25,28,33,66-70]. Before distributing the questionnaire, a readability test was conducted to validate how easily the questionnaire could be understood by respondents. The readability test was carried out both face-to-face and internet-based, using Google Meet, with 8 people who met the research criterion (ie, they all used MFAs). This readability test was carried out between February 5 and 10, 2023. We then used the results of the readability test to refine the questionnaire.

Once the questionnaire had been refined, we conducted a pilot study from February 20 to 25, 2023, aiming to measure the validity and reliability of the questionnaire by distributing it to 31 selected research respondents. The results of the pilot study were used to check the value of Cronbach α, which, in this pilot study, was 0.832, well over the required value of >0.7.

### Research Instruments

The instruments used in this study were a web-based questionnaire and semistructured interview questions. The questionnaire first asked questions regarding the demographics of the respondents, and it then presented statements regarding the research model being tested. Each of the 8 variables exclude the gender variable in the study was assessed by 3 or 4 measurement items, and each indicator was represented by a statement to which participants responded on a Likert scale ranging from 1=*strongly disagree* to 5=*strongly agree*. The questionnaire used in this study is available in Multimedia Appendix 1, and a list of the interview questions is available in Multimedia Appendix 1.

### Ethical Considerations

This research was approved by Faculty of Computer Science (approval number S-7/UN2.F11.D1.5/PPM.00.00/2024).

## Results

### Participant Demographics

We distributed the research questionnaire on the web through various social media platforms such as WhatsApp, Line, Twitter, Instagram, and Telegram. These social media platforms are widely used by Indonesians. The questionnaire distribution was carried out between February 27 and March 20, 2023. Table 1 provides a demographic summary of the respondents. Of the respondents, 75.5% (286/379) were aged between 17 and 25 years, 72.3% (274/379) were women, 25.1% (95/379) were privately employed, and 51.5% (195/379) lived in Greater Jakarta.

**Table 1:** Respondents’ demographics (n=379).

Variable		Respondents, n (%)
**Age (years)**		
	17-25	286 (75.5)
	26-35	74 (19.5)
	36-45	14 (3.7)
	>45	5 (1.3)
**Gender**		
	Woman	274 (72.3)
	Man	105 (27.8)
**Occupation**		
	College student	201 (53)
	Employee of state-owned enterprise	8 (2.1)
	High school student	15 (4)
	Privately employed	95 (25.1)
	Unemployed	19 (5)
	Entrepreneur	23 (6.1)
	Housewife	2 (0.5)
	Civil servant	3 (0.8)
	Other	13 (3.4)
**Domicile**		
	Greater Jakarta	195 (51.5)
	Java island	137 (36.1)
	Outside of Java island	47 (12.4)

After collecting both the quantitative and qualitative data, we processed the quantitative data using covariance-based structural equation modeling. Using covariance-based structural equation modeling, data processing is carried out in several stages: specification and identification of the research model, estimation of the research model, testing the feasibility of the research model, modification of the research model, and hypothesis testing.

To validate the quantitative data results, we also collected qualitative data by conducting semistructured interviews with 30 respondents. The interviews were conducted both offline and on the web and took 30 to 45 minutes each. The qualitative data analysis was carried out thematically on the basis of the defined hypotheses.

### Measurement Model

The factor loading values of all variables and indicators met the Cronbach α standard of >0.7 [71]; thus, the model feasibility test could be carried out. This study yielded average variance extracted values >0.5 as well as Cronbach α and composite reliability values >0.7 [71] (Table 2).

**Table 2:** Average variance extracted, Cronbach α, and composite reliability values.

Variable	Average variance extracted	Cronbach α	Composite reliability
Self-monitoring	0.968	0.705	0.920
Platform rewards	0.773	0.927	0.872
External influence	0.865	0.816	0.834
Social support	0.638	0.854	0.835
Confirmation of expectations	0.975	0.763	0.885
Perceived usefulness	0.709	0.888	0.848
Satisfaction	0.669	0.889	0.889
Continuance use intention	0.640	0.812	0.842

### Structural Model

Next, we tested the structural model with the goodness-of-fit criteria, which included the relative chi-square index, goodness-of-fit index, root-mean-square error of approximation, root mean square residual, normal fit index, comparative fit index, and the Tucker-Lewis Index [71]. The goodness-of-fit values are presented in Table 3, and the *R*^2^ values are shown in Table 4.

**Table 3:** Goodness-of-fit values.

Goodness-of-fit criteria	Cutoff value	Value	Description
Relative chi-square index	<2	1.956	Good fit
Goodness-of-fit index	≥0.9	0.900	Good fit
Root-mean-square residual	≤0.05	0.048	Good fit
Normal fit index	≥0.9	0.913	Good fit
Comparative fit index	≥0.9	0.955	Good fit
Tucker-Lewis Index	≥0.9	0.948	Good fit
Root-mean-square error of approximation	≤0.08	0.050	Good fit

**Table 4:** *R^2^* values.

Variable	*R^2^*	Effect size
Perceived usefulness	0.349	Weak
Satisfaction	0.511	Medium
Continuance use intention	0.714	Strong

### Hypotheses Testing

This study used a 2-tailed significance test; thus, the condition for accepting the hypothesis was *P*<.05 [71]. Table 5 presents the results of hypotheses 1 to 4 and 9 to 12, only one of which (H2) was rejected.

**Table 5:** Hypotheses testing results.

Hypothesis	Estimate (95% CI)	*P* value	Result
H1: SM^a^→PU^b^	0.319 (0.244 to 0.394)	.001	Accepted
H2: SS^c^→PU	0.060 (−0.019 to 0.143)	.12	Rejected
H3: PR^d^→PU	0.136 (0.044 to 0.219)	.007	Accepted
H4: EI^e^→PU	−0.101 (−0.166 to −0.033)	.006	Accepted
H9: COE^f^→PU	0.323 (0.251 to 0.388)	.001	Accepted
H10: COE→satisfaction	0.541 (0.435 to 0.632)	.002	Accepted
H11a: PU→CUI^g^	0.280 (0.200 to 0.363)	.002	Accepted
H11b: PU→satisfaction	0.218 (0.069 to 0.355)	.003	Accepted
H12: Satisfaction→CUI	0.683 (0.560 to 0.813)	.001	Accepted

^a^SM: self-monitoring.

^b^PU: perceived usefulness.

^c^SS: social support.

^d^PR: platform rewards.

^e^EI: external influence.

^f^COE: confirmation of expectations.

^g^CUI: continuance usage intention.

According to Awang [72], the test for moderation is not significant when the difference in chi-square values between the constrained model and the unconstrained model is <3.84. Table 6 presents a summary of the results of the hypothesis testing using the moderating effect of gender. On the basis of the difference in the chi-square values between the constrained model and the unconstrained model, it can be concluded that all difference values were >3.84 and therefore meet the requirements for calculating the significance of the moderating effect, meaning that H5, H6, H7, and H8 were all accepted.

**Table 6:** Summary of moderating variable hypothesis testing, with gender as the moderating effect.

Path	Chi-square constrained model	Chi-square unconstrained model	Difference (*df*)	Result
SM^a^→PU^b^	1318.671	1043.151	275.520	H5 accepted
SS^c^→PU	1627.823	1043.151	584.672	H6 accepted
PR^d^→PU	1395.076	1043.151	351.925	H7 accepted
EI^e^→PU	1796.875	1043.151	753.724	H8 accepted

^a^SM: self-monitoring.

^b^PU: perceived usefulness.

^c^SS: social support.

^d^PR: platform rewards.

^e^EI: external influence.

### Qualitative Interviews and Validity of the Hypotheses

This research shows that the incentives offered in MFAs in the form of SM (eg, distance walked or run, number of calories expended, time taken, and heart rate) influence users’ motivation to undertake PA. The acceptance of H1 is thus in accordance with the findings of Yin et al [17] and Stragier et al [73]. Yin et al [17] state that the user’s PA level correlates positively with the amount of SM they do. The majority of interviewees felt that the SM feature provided encouragement for their PA:

So I feel happy because I have exercised, more enthusiasm.

Interviewee 6

In addition, the interviewees believed that MFAs documented or tracked their progress in PA, which helped them to maintain or even improve their exercise consistency:

So that I can compare with previous progress and so that in the future I can look back at my history. Like pace, I also remember what date I did sport.

Interviewee 9

An example of a feature that can be implemented is one that displays a summary of the user’s performance while exercising, together with visualizations in the form of trends and graphs. Some apps also display comments that describe the user’s sports activity performance, based on their activity level. Users can take advantage of these insights to increase their PA levels in their next sports activity.

However, H2 was rejected in this study. H2’s rejection aligns with the findings of Sun and Jiang [74] and Kim et al [75]. According to Kim et al [75], social comparison and the user’s level of PA are not directly connected. Social comparison here is defined as the relationship between the level of PA and the variable self-efficacy, or a person’s belief in their own capabilities [75]. The rejection of H2 indicates that the community or social ecosystem around MFA users does not have a significant impact on motivating the users to exercise. On the basis of the interviews, the SS feature in the app does not have an important effect on PA levels because users do not feel compelled to exercise when using the SS feature:

There is no motivation from the engagement side, more from tracking my own progress.

Interviewee 9

In addition, nearly one-third of the interviewees (9/30, 30%) admitted that they used the SS feature only to document sports activities that had already been completed.

H3 was accepted in this study. The acceptance of H3 aligns with the studies by Bojd et al [41], Payne et al [42], Plangger et al [13], Goes et al [76], and Hamari and Koivisto [77]. Bojd et al [41] found that the gamification element in MFAs can provide two types of information: (1) the user’s PA progress and (2) a comparison of the user’s PA with that of other users. Furthermore, when MFA users are able to observe their progress, they feel more satisfied and recognize their own PA competency, which will drive higher user competency satisfaction and behavioral motivation [13,42]. Goes et al [76] and Hamari and Koivisto [77] also highlight the gamification element in MFA, which tracks the user’s effort, progress, and achievement of personal goals. According to Goes et al [76], the public nature of user-acquired gamification elements, such as levels, badges, or leaderboards, can generate users’ social status on the MFA platform, which encourages social comparison and competitive motivation among users. On the basis of the interviews, MFA users want to take part in challenges (an example of implementing gamification) on the app because they want to obtain limited edition rewards and measure their own capabilities in sports activities:

Gamification keeps me motivated and helps me see my activities historically during physical activity based on the badge I have earned.

Interviewee 21

Furthermore, the interviewees acknowledged that the rewards they obtain can be used as a benchmark of their capacity in the sports activity against which to build new achievements:

I feel happy when I get an achievement because it shows an improvement in my sport. Even though I don’t I have specifically targeted certain achievements, but if I can surpass the previous achievements, it means that my sport has improved. The goals that I have set are higher than before.

Interviewee 17

Therefore, it would be better if the MFA included challenges that were personalized as well as recommendations that were based on the user’s type of sports activity, the user’s sports activity goals, and the user’s own sports activity history. An example of such a feature could be that, based on the user’s history, if they have only managed to run a distance of 3 km, then, to improve their performance, other MFA users could recommend a 4-km challenge.

Huang [28] found that sponsor characteristics play an important role in triggering user behavior. Sponsorship referred to circumstances where the use of a sponsor’s product occurred naturally as part of a sponsored event [78]; for example, with an MFA whose function is to promote PA, sponsorship of athletic apparel would be perceived as highly congruent, whereas sponsorship of a cold remedy would reflect low congruence. The H4 finding is in line with the study by Yang et al [79], who stated that the level of involvement of a brand produces a positive association with the brand and strengthens the positive effect of an evaluation impacting one’s behavioral intention toward an app. From the interviews, it was found that interviewees were encouraged to take part in a challenge or activity if the activity was associated with the party (public figure, company, etc) that organized it:

For a club other than Strava, I think it’s cool if you participate, for example, it’s like unique. There’s definitely a challenge made by Strava every month, so it’s not as special as other clubs. The limited edition is more about Heart Month, New Year, and others. I want to take part because it would be a shame if I didn’t follow.

Interviewee 9

We found that not many interviewees took advantage of EI incentives, but those who did participate focused more on the challenges than on the organizers or the external community. If a user felt capable of taking part in a challenge, they would try to do so:

Actually, I see from the challenge, if I feel capable, then I want to join.

Interviewee 7

Thus, we argue that it would be better if MFA developers or providers developed challenges for their apps that are created by communities, organizations, and figures with high functional congruence.

With regard to H5, H6, H7, and H8, the results show that, in every case, gender has a moderating effect on the relationship between the variables investigated. This study showed that gender influences the relationship between SM and PU (H5). These results are supported by the studies by Gabriel and Gardner [46] and Sun et al [51], who found that men tend to make decisions based on rationality, while women tend to be more perceptual. According to Gabriel and Gardner [46] and Sun et al [51], men are generally more focused on personal goals that demonstrate their individual performance and abilities, while women are usually less conscious of their own goals and performance. This finding is supported by van Elburg et al [80], who state that men focus more on practical goals and achieving goals when using an mHealth app. We found that our female interviewees usually used the metrics in MFAs for tracking their PA only as monitoring information:

I only look at the pulse.

Interviewee 1

However, the men usually used these metrics as targets for self-development:

To find out whether in sports we have reached the desired target or not. On the other hand, if our sports performance is good, this can also be seen through the information displayed on Apple Watch. Thus, the Apple Watch can be a helpful tool in determining whether our performance has reached the expected level or not.

Interviewee 12

Moreover, this study found that gender influenced the extent to which SS incentives affected users’ PU (H6). The results of the interviews showed that most female respondents felt more motivated by their social community or by the SS feature provided in the MFA they used. By contrast, the male users used the SS feature, such as sharing their sports activity progress, for personal documentation purposes:

Just so you know. Only for review, not to share with other friends.

Interviewee 28

Other male respondents stated that this was the case simply because the app posted their activity automatically*:*

Because it has to be posted on the Strava application.

Interviewee 8

Many male respondents had never used this feature, indicating their lack of interest in the SS feature:

I have never tried it.

Interviewee 18

However, the female respondents all expressed interest in the SS feature available in MFAs and felt more motivated to exercise due to this feature:

I also become motivated to exercise when I see my friends after posting their sports results.

Interviewee 11

Some of the female respondents commented that the SS feature of MFAs motivated them to exercise by creating a sense of competition:

If I just wake up in the morning and get a notification that my friend has finished exercising, I feel left behind because I just woke up but he has finished exercising. Section it motivates, really.

Interviewee 9

Relatedly, Li et al [33] found that women pay greater attention to social relations and are more willing to accept support from those around them. By contrast, Leong et al [54] found that men usually ignore external advice or support due to their sense of independence. These findings are supported by Yin et al [17], who found that SS had a more positive effect on PA in women than in men.

This study also showed that gender influences the relationship between platform rewards and PU (H7). The interviews showed that male respondents were generally more motivated by the challenges, badges, and awards offered by the MFA they were using:

Makes me more enthusiastic for the next run, and I use it to keep track of whether I should improve or maintain, for example, I can rank third so I feel I have to improve my performance.

Interviewee 16

However, the female respondents usually followed or used this feature only for their own satisfaction and without specific targets or motivations:

There is no specific goal to get rewards, but I feel happy and proud of myself if I get them.

Interviewee 13

According to Yin et al [17], in the context of PA, men usually pay more attention to meeting their needs for autonomy and competence, such as badges, awards, and so on. This was also demonstrated by Vilela and Nelson [52], who stated that men tend to be more aggressive, pragmatic, and self-oriented. Therefore, they are motivated by the need for achievement when using information system products [52]. Similar findings were identified by Forman et al [81], who showed that the gamification element has a more positive effect on men than on women by arousing their competitive and achievement-oriented motivation. Brandts et al [82] also support this finding and explain that task-based goal setting increases task completion and performance only for men.

This study also showed that gender affected the impact of EI on the user’s PU of MFAs (H8). The results of the interviews confirmed that there are 2 main reasons a person will participate in PA supported by the MFA: the match between the organizer of the activity and the user and the match between the user’s capabilities and the activity or challenge created. Comparing these 2 reasons, we found that the women were more likely to do something because of a match with the organizers, in contrast to the men, who usually focused more on their own ability to participate in an activity:

If Strava doesn’t have motivation, if it’s a club other than in my opinion, Strava is cool if you join, it’s like unique. What Strava makes is there every month, so it’s not as special as other clubs to participate on Strava.

Interviewee 29

According to Huang [28] and Yang et al [79], the reason female MFA users participate in sports activities is that they experience a *special* feeling because these sports activities are created by a special club. Huang [28] and Yang et al [79] explain that the sponsorship characteristics of a sports activity and high brand involvement play important roles in triggering the behavioral intention of MFA users and their behavior in general. H8 is also supported by the findings of Weman Josefsson et al [56], who explain that men tend to be more influenced by winning rewards than women, who tend to participate more for autonomous and social reasons.

Furthermore, H9 was confirmed in this study. The acceptance of H9 is in accordance with previous research conducted by Bhattacherjee [38], Huang et al [15], Chiu et al [25], Wang et al [9], Cai et al [83], and Wu et al [66]. Wu et al [66] found that PU and user satisfaction are directly influenced by confirmation of expectations, namely, the realization of the expected benefits of using mHealth. This result is supported by Chiu et al [25], who state that PU of the MFA is reflected in the user’s enhanced exercise capacity and satisfaction, as evidenced by their increased enjoyment of exercising. Thus, it is to be expected that, after the initial experience, the confirmation level of the user’s expectations will have a positive effect on their PU [9,15,38,83]. One of the expectations of a respondent who used an MFA was that they would experience changes and improvements in their PA or exercise, and these expectations were indeed successfully confirmed:

Because when I want to download Strava I want to be diligent in exercising, and it is proven that I exercise more often because I can track my sports progress.

Interviewee 23

H10 was also accepted by this study, and this result is in accordance with the studies by Bhattacherjee [38], Huang et al [15], Chiu et al [25], Wang et al [9], Cai et al [83], and Wu et al [66]. Wang et al [9] found that confirmation of expectations positively affects user satisfaction with IT products and services. The results of the interviews confirmed that interviewees felt satisfaction when using MFAs:

From a user point of view, everything has been fulfilled in my opinion. What I need so far has been achieved.

Interviewee 24

In my opinion, the features are quite complete, because that’s all I really need. The application also provides a reminder if you have passed one day without exercising and automatically arranges for the workout that can be fulfilled the next day to be even tougher.

Interviewee 10

This study also showed that PU influences CUI. Acceptance of H11a is in accordance with the studies by Bhattacherjee [38], Huang et al [15], Chiu et al [25], Huang and Ren [26], Wang et al [9], Cai et al [83], Wu et al [66], and Cho et al [24]. Cho et al [24] reported that, in the context of MFAs, perceived benefits were associated with managing health-related information. The interviews confirmed that interviewees would continue using the MFAs if they helped them to be more active in their exercising, and they could track their sports activity progress effectively:

I will continue to use it because in my opinion it is also effective and looks simple.

Interviewee 10

As long as device is connected to the Apple Watch, will still use it. The ability to track different types of exercise separately is one of the advantages of the Apple Watch. This makes me still choose to use the Apple Watch in the future, as long as it meets my sporting needs.

Interviewee 12

The study’s acceptance of H11b is in accordance with the studies by Bhattacherjee [38], Huang et al [15], Chiu et al [25], Wang et al [9], Cai et al [83], and Wu et al [66]. Cai et al [83] explain that PU is reflected in user satisfaction when exercising using an MFA. The more benefits users obtain from the MFA, the greater their satisfaction [25]. Wang et al [9] also found that satisfaction was a partial mediator between CUI and PU. We found that the level of user satisfaction with an MFA was based not only on its meeting users’ sports activity expectations but also on the convenience and effectiveness of the features, the user interface, and the user experience that supported the user’s sports activities:

I will continue to use Strava, because I am comfortable with Strava.

Interviewee 14

I will continue to use it, because in my opinion it is also effective and the appearance is not a hassle.

Interviewee 10

What makes me satisfied is the user interface, which is easy to use, and the user experience is simple.

Interviewee 19

Finally, the effect of satisfaction on CUI was confirmed in this study. The acceptance of H12 is in accordance with the studies by Bhattacherjee [38], Huang et al [15], Chiu et al [25], Wang et al [9], Cai et al [83], and Wu et al [66]. Wu et al [66] found that satisfied users are more likely to continue using an app because dissatisfied users can easily switch to other mHealth technologies. User satisfaction is an important determinant of the postadoption behavior of users of IT products and services [38]. This is supported by Chiu et al [25], who state that user satisfaction with the use of IT products and services is very important for fostering long-term use of IT. The main reason for user satisfaction with an MFA is that the features are complete and meet user needs, with the result that they come to depend on the MFA for their exercise routines:

Because I really like it and I have become very dependent on this application for sports. I don’t want to exercise if there is no access to this application.

Interviewee 9

This application has fulfilled my daily needs.

Interviewee 15

## Discussion

### Principal Findings

The findings from this study extend previous research by examining the incentive system in MFAs [17,26] and the use of the ECT in the context of mHealth [25,33,38,62,66]. It also expands the understanding of the moderating effect of gender on incentive-based systems [68]. We found that MFAs and the incentives they offer have a strong influence on users’ sports activity behaviors and on their intention to continue using the app. The results of this study indicate that the most influential feature of an MFA is the SM incentive feature. MFA users often do not feel like exercising or engaging in PA if the activity is not being tracked by their app. The SM feature was also found to have a greater impact on male users than on female users. This finding regarding gender differs from the results of a study by Yin et al [17], who stated that no gender trend was evident in the effectiveness of the SM feature. Furthermore, in contrast to the study by Yin et al [17], we found that SS had little effect on the PA of MFA users. The results of the qualitative interviews indicate that this is because the social circle of Indonesian MFA users is relatively small, and this small social circle affects the effectiveness of the SS feature.

MFA service providers should evaluate how different app features impact users of different genders to effectively motivate users to keep using their app in the long term. In addition, users feel more satisfied when their expectations regarding the use of an app are met. App developers can increase the PU of their MFA by using the users’ social communities (eg, by creating social profile features, group exercises, sporting events organized by recognized organizations or communities, and personalized challenges or awards based on the user’s sports activity history). App developers can improve the accuracy of the tracking feature, whether through a smartphone or a smartwatch, with the goal of providing users with more in-depth statistics and data. For user convenience, app providers should also develop tracking features that start automatically.

### Limitations

The respondents to both the quantitative and qualitative studies were predominantly aged 17 to 25 years and female; thus, other moderating variables could be considered in a future study. The weak effect size for PU in Table 4 indicates that the differences or relationships between some variables were not significant. This suggests that there are other variables that might influence PU, which were not considered in this study. In future research, another variable that could be considered is PA. This could serve as a metric to determine whether using MFAs with specific incentives increases users’ PA [17].

### Conclusions

The results of the study show that SM, platform rewards, and EI can all influence the PU of MFAs. However, no relationship was found between SS and the PU of MFAs. Indonesians generally consider MFAs to be useful because these apps allow them to track their sports activities and also offer rewards and awards. The confirmation of a user’s initial expectations also affects their perceptions of the usefulness of MFAs. PU and confirmation of expectations also affect user satisfaction with MFAs, which in turn influences the user’s desire to continue using the MFA. In addition, gender was shown to influence user behavior when using MFAs. In future research, the scope of EI incentives could be expanded by considering financial reasons for exercising, other people’s recommendations, and job demands, among other factors. We suggest considering tangible benefits as additional incentives to determine whether quantifiable benefits, such as assets or money, can increase a person’s motivation to exercise.

## Appendix

Multimedia Appendix 1 Study questionnaire and interview questions.

## Abbreviations

CUI: continuance usage intention
ECT: expectation-confirmation theory
EI: external influence
MFA: mobile fitness app
mHealth: mobile health
PA: physical activity
PU: perceived usefulness
SDT: self-determination theory
SM: self-monitoring
SS: social support

## Data Availability

The data sets generated and analyzed during this study are not publicly available due to a lack of authorization to share these data.

## References

[1] (2022). Physical activity. World Health Organization.

[2] (2022). The global status report on physical activity. World Health Organization.

[3] Global recommendations on physical activity for health. World Health Organization.

[4] (2022). Consumer sentiment in Indonesia during the coronavirus crisis. McKinsey & Company.

[5] (2023). Fitness apps- Indonesia. Statista.

[6] Edney SM, Olds TS, Ryan JC, Vandelanotte C, Plotnikoff RC, Curtis RG, Maher CA (2020). A social networking and gamified app to increase physical activity: cluster RCT. Am J Prev Med.

[7] Mao X, Zhao X, Liu Y (2020). mHealth app recommendation based on the prediction of suitable behavior change techniques. Decis Support Syst.

[8] Tu R, Hsieh P, Feng W (2021). Walking for fun or for “likes”? The impacts of different gamification orientations of fitness apps on consumers’ physical activities. Sport Manag Rev.

[9] Wang Y, Wang Y, Greene B, Sun L (2020). An analysis and evaluation of quality and behavioral change techniques among physical activity apps in China. Int J Med Inform.

[10] Baker RC, Kirschenbaum DS (1993). Self-monitoring may be necessary for successful weight control. Behav Ther.

[11] Boutelle KN, Kirschenbaum DS (1998). Further support for consistent self-monitoring as a vital component of successful weight control. Obes Res.

[12] Yan L, Tan Y (2018). Good intentions, bad outcomes: the effects of mismatches between social support and health outcomes in an online weight loss community. Prod Oper Manag.

[13] Plangger K, Campbell C, Robson K, Montecchi M (2022). Little rewards, big changes: using exercise analytics to motivate sustainable changes in physical activity. Inf Manag.

[14] Uetake K, Yang N (2020). Inspiration from the “biggest loser”: social interactions in a weight loss program. Mark Sci.

[15] Huang CK, Chen CD, Liu YT (2019). To stay or not to stay? Discontinuance intention of gamification apps. Inform Technol Peopl.

[16] Zhu Y, Wang R, Zeng R, Pu C (2022). Does gender really matter? Exploring determinants behind consumers' intention to use contactless fitness services during the COVID-19 pandemic: a focus on health and fitness apps. Internet Res.

[17] Yin Q, Li L, Yan Z, Guo C (2021). Understanding the effects of self-peer-platform incentives on users' physical activity in mobile fitness apps: the role of gender. Inform Technol Peopl.

[18] Miller AS, Cafazzo JA, Seto E (2016). A game plan: gamification design principles in mHealth applications for chronic disease management. Health Informatics J.

[19] de Zambotti M, Claudatos S, Inkelis S, Colrain IM, Baker FC (2015). Evaluation of a consumer fitness-tracking device to assess sleep in adults. Chronobiol Int.

[20] Sama PR, Eapen ZJ, Weinfurt KP, Shah BR, Schulman KA (2014). An evaluation of mobile health application tools. JMIR Mhealth Uhealth.

[21] Willms A, Rhodes RE, Liu S (2023). Effects of mobile-based financial incentive interventions for adults at risk of developing hypertension: feasibility randomized controlled trial. JMIR Form Res.

[22] Bo Y, Liu QB, Tong Y (2023). The effects of adopting mobile health and fitness apps on hospital visits: quasi-experimental study. J Med Internet Res.

[23] Yuan S, Ma W, Kanthawala S, Peng W (2015). Keep using my health apps: discover users' perception of health and fitness apps with the UTAUT2 model. Telemed J E Health.

[24] Cho H, Chi C, Chiu W (2020). Understanding sustained usage of health and fitness apps: incorporating the technology acceptance model with the investment model. Technol Soc.

[25] Chiu W, Cho H, Chi CG (2020). Consumers' continuance intention to use fitness and health apps: an integration of the expectation–confirmation model and investment model. Inform Technol Peopl.

[26] Huang G, Ren Y (2020). Linking technological functions of fitness mobile apps with continuance usage among Chinese users: moderating role of exercise self-efficacy. Comput Human Behav.

[27] Teng X, Bao Z (2022). Factors affecting users’ stickiness of fitness apps: an empirical study based on the S-O-R perspective. Int J Sports Mark Spons.

[28] Huang G (2021). Does warm glow promote physical activity? Examining the relative effectiveness of self-benefiting versus other-benefiting incentives in motivating fitness app use by corporate sponsorship programs. Health Commun.

[29] Pearson E, Prapavessis H, Higgins C, Petrella R, White L, Mitchell M (2020). Adding team-based financial incentives to the Carrot Rewards physical activity app increases daily step count on a population scale: a 24-week matched case control study. Int J Behav Nutr Phys Act.

[30] Lemola S, Gkiouleka A, Read B, Realo A, Walasek L, Tang NK, Elliott MT (2021). Can a 'rewards-for-exercise app' increase physical activity, subjective well-being and sleep quality? An open-label single-arm trial among university staff with low to moderate physical activity levels. BMC Public Health.

[31] Ryan RM, Deci EL (2000). Self-determination theory and the facilitation of intrinsic motivation, social development, and well-being. Am Psychol.

[32] Teixeira PJ, Carraça EV, Markland D, Silva MN, Ryan RM (2012). Exercise, physical activity, and self-determination theory: a systematic review. Int J Behav Nutr Phys Act.

[33] Li J, Liu X, Ma L, Zhang W (2019). Users' intention to continue using social fitness-tracking apps: expectation confirmation theory and social comparison theory perspective. Inform Health Soc Care.

[34] Bandura A (1991). Social cognitive theory of self-regulation. Organ Behav Hum Decis Process.

[35] Davis FD, Bagozzi RP, Warshaw PR (1989). User acceptance of computer technology: a comparison of two theoretical models. Manag Sci.

[36] Barkley JE, Lepp A, Santo A, Glickman E, Dowdell B (2020). The relationship between fitness app use and physical activity behavior is mediated by exercise identity. Comput Human Behav.

[37] Schoeppe S, Alley S, Van Lippevelde W, Bray NA, Williams SL, Duncan MJ, Vandelanotte C (2016). Efficacy of interventions that use apps to improve diet, physical activity and sedentary behaviour: a systematic review. Int J Behav Nutr Phys Act.

[38] Bhattacherjee A (2001). Understanding information systems continuance: an expectation-confirmation model. MIS Q.

[39] Wang Q, Egelandsdal B, Amdam GV, Almli VL, Oostindjer M (2016). Diet and physical activity apps: perceived effectiveness by app users. JMIR Mhealth Uhealth.

[40] Chen Y, Pu P (2014). Healthy together: exploring social incentives for mobile fitness applications. Proceedings of the 2nd International Symposium of Chinese CHI.

[41] Bojd B, Song X, Tan Y, Yan X (2018). Gamified challenges in online weight-loss communities. SSRN Journal. Preprint posted online April 25, 2018.

[42] Payne HE, Lister C, West JH, Bernhardt JM (2015). Behavioral functionality of mobile apps in health interventions: a systematic review of the literature. JMIR Mhealth Uhealth.

[43] Bollók S, Takács J, Kalmár Z, Dobay B (2011). External and internal sport motivations of young adults. Biomed Hum Kinet.

[44] Koo S, Byon KK, Baker TA (2014). Integrating event image, satisfaction, and behavioral intention: small-scale marathon event. Sport Mark Q.

[45] Low XT, Pyun DY (2016). Consumers' perceived functions of and attitude toward corporate sponsors of small-scale amateur sporting events. Event Manag.

[46] Gabriel S, Gardner WL (1999). Are there "his" and "hers" types of interdependence? The implications of gender differences in collective versus relational interdependence for affect, behavior, and cognition. J Pers Soc Psychol.

[47] Ryan RM, Williams GC, Heather H, Deci EL (2009). Self-determination theory and physical activity: the dynamics of motivation in development and wellness. Hellenic J Psychol.

[48] Weisskopf WA (1967). The duality of human existence: an essay on psychology and religion by David Bakan. Am J Sociol.

[49] Spence JT, Helmreich RL (1978). Masculinity and femininity: their psychological dimensions, correlates, and antecedents. Psychol Women Q.

[50] Kimbrough AM, Guadagno RE, Muscanell NL, Dill J (2013). Gender differences in mediated communication: women connect more than do men. Comput Human Behav.

[51] Sun Y, Lim KH, Jiang C, Peng JZ, Chen X (2010). Do males and females think in the same way? An empirical investigation on the gender differences in web advertising evaluation. Comput Human Behav.

[52] Vilela AM, Nelson MR (2013). Testing the selectivity hypothesis in cause-related marketing among generation Y: [when] does gender matter for short- and long-term persuasion?. J Mark Comm.

[53] Sun H, Zhang P (2006). The role of moderating factors in user technology acceptance. Int J Hum Comput Stud.

[54] Leong LY, Ooi KB, Chong AY, Lin B (2013). Modeling the stimulators of the behavioral intention to use mobile entertainment: does gender really matter?. Comput Human Behav.

[55] Venkatesh V, Thong JY, Xu X (2012). Consumer acceptance and use of information technology: extending the unified theory of acceptance and use of technology. MIS Q.

[56] Weman Josefsson K, Johnson U, Lindwall M (2018). Short report: moderations in exercise motivation - gender and age moderates the relations of motivation quality and exercise behavior. Health Psychol Behav Med.

[57] Albashrawi M, Motiwalla L (2017). When IS success model meets UTAUT in a mobile banking context: a study of subjective and objective system usage. Proceedings of the 30th Annual Workshop of the Swedish Artificial Intelligence Society.

[58] Susanto A, Chang Y, Ha Y (2016). Determinants of continuance intention to use the smartphone banking services. Ind Manage Data Syst.

[59] Oghuma AP, Libaque-Saenz CF, Wong SF, Chang Y (2016). An expectation-confirmation model of continuance intention to use mobile instant messaging. Telemat Inform.

[60] Festinger L (1957). A Theory of Cognitive Dissonance.

[61] Bhattacherjee A (2001). An empirical analysis of the antecedents of electronic commerce service continuance. Decis Support Syst.

[62] Hsu CL, Lin JC (2015). What drives purchase intention for paid mobile apps? – An expectation confirmation model with perceived value. Electron Commer Res Appl.

[63] Ambalov IA (2018). A meta-analysis of IT continuance: an evaluation of the expectation-confirmation model. Telemat Inform.

[64] Dehghani M, Kim KJ, Dangelico RM (2018). Will smartwatches last? factors contributing to intention to keep using smart wearable technology. Telemat Inform.

[65] Nascimento B, Oliveira T, Tam C (2018). Wearable technology: what explains continuance intention in smartwatches?. J Retail Consum Serv.

[66] Wu C, Zhou Y, Wang R, Huang S, Yuan Q (2022). Understanding the mechanism between IT identity, IT mindfulness and mobile health technology continuance intention: an extended expectation confirmation model. Technol Forecast Soc Change.

[67] Vinnikova A, Lu L, Wei J, Fang G, Yan J (2020). The use of smartphone fitness applications: the role of self-efficacy and self-regulation. Int J Environ Res Public Health.

[68] Sharma P, Sivakumaran B, Marshall R (2010). Impulse buying and variety seeking: a trait-correlates perspective. J Bus Res.

[69] Sheikh Z, Yezheng L, Islam T, Hameed Z, Khan IU (2019). Impact of social commerce constructs and social support on social commerce intentions. Inform Technol Peopl.

[70] Ng JY, Ntoumanis N, Thøgersen-Ntoumani C, Deci EL, Ryan RM, Duda JL, Williams GC (2012). Self-determination theory applied to health contexts: a meta-analysis. Perspect Psychol Sci.

[71] Hair Jr JF, Matthews LM, Matthews RL, Sarstedt M (2017). PLS-SEM or CB-SEM: updated guidelines on which method to use. Int J Multivar Data Anal.

[72] Awang Z (2015). SEM Made Simple: A Gentle Approach to Learning Structural Equation Modelling.

[73] Stragier J, Vanden Abeele M, Mechant P, De Marez L (2016). Understanding persistence in the use of online fitness communities: comparing novice and experienced users. Comput Human Behav.

[74] Sun M, Jiang LC (2022). Linking social features of fitness apps with physical activity among Chinese users: evidence from self-reported and self-tracked behavioral data. Inf Process Manage.

[75] Kim HM, Cho I, Kim M (2022). Gamification aspects of fitness apps: implications of mHealth for physical activities. Int J Hum Comput Interact.

[76] Goes PB, Guo C, Lin M (2016). Do incentive hierarchies induce user effort? Evidence from an online knowledge exchange. Inf Syst Res.

[77] Hamari J, Koivisto J (2015). “Working out for likes”: an empirical study on social influence in exercise gamification. Comput Human Behav.

[78] Pappu R, Cornwell TB (2014). Corporate sponsorship as an image platform: understanding the roles of relationship fit and sponsor–sponsee similarity. J Acad Mark Sci.

[79] Yang J, Kanthawala S, Joo E, Kononova A (2021). Can brand sponsorship increase download intention for mHealth apps? The role of issue relevance, brand involvement, and perceived app quality. J Promot Manag.

[80] van Elburg FR, Klaver NS, Nieboer AP, Askari M (2022). Gender differences regarding intention to use mHealth applications in the Dutch elderly population: a cross-sectional study. BMC Geriatr.

[81] Forman EM, Manasse SM, Dallal DH, Crochiere RJ, Berry MP, Butryn ML, Juarascio AS (2021). Gender differences in the effect of gamification on weight loss during a daily, neurocognitive training program. Transl Behav Med.

[82] Brandts J, El Baroudi S, Huber SJ, Rott C (2021). Gender differences in private and public goal setting. J Econ Behav Organ.

[83] Cai J, Zhao Y, Sun J (2021). Factors influencing fitness app users’ behavior in China. Int J Hum Comput Interact.

