# Supplementary material for: The Influence of Incentive-Based Mobile Fitness Apps on Users’ Continuance Intention With Gender Moderation Effects: Quantitative and Qualitative Study
Source: JMIR Hum Factors. 2024 Jun 5;11:e50957. doi: 10.2196/50957 (PMC11187517; doi:10.2196/50957)
Supplement: Multimedia Appendix 1 [file humanfactors_v11i1e50957_app1.docx]

# Appendix A. Questionnaire

| Code | Measurement Items | Reference |
| --- | --- | --- |
| SM1 | I watch for signs of progress (steps, distance, time, etc.) as I stay physically active. | Vinnikova et al. [67] |
| SM2 | I monitor myself to see if I am meeting my goals for physical activity. |  |
| SM3 | I can adjust my behavior in physical activity to meet the requirements in MFA that I find myself in. | Sharma et al. [68] |
| SM4 | Once I know what my needs are from MFA, it's easy for me to regulate my physical activity accordingly. |  |
| SS1 | When faced with difficulties in my PA, some people on MFA comforted and encouraged me. | Sheikh et al. [69] |
| SS2 | When faced with difficulties in my PA, some people on MFA listened to me and let me talk about my feelings. |  |
| SS3 | When faced with difficulties in my PA, some people on MFA expressed interest and concern in my well-being. |  |
| SS4 | I like interacting with others on the MFA community. | Zhu et al. [16] |
| PR1 | Gamification in MFA (badges, reward, points, leaderboard, challenge, etc) makes me want to use it. | Huang et al. [15] |
| PR2 | I enjoy using the gamification in MFA. |  |
| PR3 | I feel relaxed about using the gamification in MFA. |  |
| PR4 | The use of gamification in MFA makes me feel good. |  |
| EI1 | I do physical activity with MFAs because my relatives/friends/physicians says I should. |  |
| EI2 | I do physical activity with MFAs for a cause (Donation, Campaigns, Sustainable Movement, etc.). | Ng et al. [70] |
| EI3 | I do physical activity with MFAs to contribute to my company's Corporate Social Responsibility (CSR). | Huang [28] |
| EI4 | I do physical activity with MFA because of its sponsors. | Ng et al. [70] |
| PU1 | Using MFA improves my performance in physical activity. | Cho et al. [24] |
| PU2 | Using MFA enhances my effectiveness in doing physical activity. |  |
| PU3 | Using MFA increases my productivity in doing physical activity. |  |
| PU4 | Using MFA is useful for doing physical activity. |  |
| CON1 | My physical activity goal can always be achieved. | Li et al. [33] |
| CON2 | I can always achieve the desired ranking of physical activity among my buddies. |  |
| CON3 | My actual physical activity meets or exceeds my goal. |  |
| CON4 | Overall, most of my expectations from using MFA were confirmed. |  |
| SAT1 | I feel satisfied about my overall experience using this MFA(s). | Chiu et al. [25] |
| SAT2 | I feel pleased about my overall experience using this MFA(s). |  |
| SAT 3 | My choice to use this MFA is a wise one. |  |
| SAT4 | I feel delighted about my overall experience using the gamification app MFA(s). | Wu et al. [66] |
| CUI1 | I intend to continue using MFA in the future. | Cho et al. [24] |
| CUI2 | I will keep using MFA as regularly as I do now. |  |
| CUI3 | I will always try to use MFA in my daily life. |  |
| CUI4 | I will recommend MFA to other people. | Li et al. [33] |

**Appendix B. Interview Questions**

| No | Questions |
| --- | --- |
|  |  |
| 1 | What are the Mobile Fitness Applications you use? |
| 2 | Why do you use those applications? |
| 3 | How long have you been using said MFA? |
| 4 | Do you monitor your progress through features of the MFA you use? |
| 5 | Why do you want to use those features? |
| 6 | How do you adjust your physical activity to achieve your target in the MFA you use? |
| 7 | Is there any specific tool that triggers you to use the tracking feature in your MFA? (e.g. miles, calories, time, etc) |
| 8 | Does your MFA have any social support features? (posts, likes, comments, etc.) |
| 9 | Why do you want to use that social support feature? |
| 10 | How often do you use that social support feature? (Once a week or once a month) |
| 11 | Is there any specific tool that triggers you to use the social support feature in your MFA? (e.g. followers, likes, engagement, etc) |
| 12 | Does your MFA have any platform rewards features? (daily goals, challenges, awards, etc.) |
| 13 | Why do you want to use that platform rewards feature? |
| 14 | How often do you use that platform rewards feature? |
| 15 | Is there any specific tool that triggers you to use the platform rewards feature in your MFA? (e.g. to achieve daily goals, rewards, trophies, etc.) |
| 16 | Does your MFA have any external influence features? (e.g. community challenge, campaign, charity marathon, etc.) |
| 17 | How often does an external influence feature trigger you to exercise? |
| 18 | Is there any specific reason that triggers you to use an external influence feature? (e.g. bonus or incentives, personal satisfaction, etc.) |
| 19 | Do you feel like features in MFA (SM, SS, PR, or EI) is useful for your physical activity? If yes, which feature influences you the most? |
| 20 | How do MFA features help you in increasing your exercise productivity? Can you explain which feature helps you the most? |
| 21 | What is the MFA feature you use most? Why? |
| 22 | Is there any difference between your expectation and actual use of MFA? |
| 23 | What are some of the downsides of the MFA you use? Is there any feature you think should have been provided? |
| 24 | Would you continue to use MFA when you are exercising? |
| 25 | Would you recommend the MFA you are currently using to others? |
| 26 | Do you notice any difference in behaviours between female and male MFA users? |
| 27 | In your opinion, what must an MFA have for it to be used continuously by its users? |
